# The associations of job strain and leisure-time physical activity with the risk of hypertension: the population-based Midlife in the United States cohort study

**DOI:** 10.4178/epih.e2022073

**Published:** 2022-09-07

**Authors:** Xinyue Liu, Timothy A. Matthews, Liwei Chen, Jian Li

**Affiliations:** 1 Department of Epidemiology, Fielding School of Public Health, University of California Los Angeles, Los Angeles, CA, USA; 2 Department of Environmental Health Sciences, Fielding School of Public Health, University of California Los Angeles, Los Angeles, CA, USA; 3 School of Nursing, University of California Los Angeles, Los Angeles, CA, USA

**Keywords:** Exercise, Hypertension, Job strain, Occupational stress, Physical activity

## Abstract

**OBJECTIVES:**

Job strain is positively associated with incident hypertension, while increasing leisure-time physical activity (LTPA) can reduce incident hypertension. However, the joint associations of job strain and LTPA with incident hypertension among United States workers have yet to be investigated. This study examined the independent and joint associations of job strain and LTPA with incident hypertension.

**METHODS:**

This prospective cohort study (n=1,160) utilized data from the population-based Midlife in the United States study. The associations of job strain and LTPA at baseline with incident hypertension during follow-up were examined using Cox proportional hazards models. High job strain was derived from a combination of high job demands and low job control, and high LTPA was defined as engagement in moderate or vigorous LTPA at least once per week.

**RESULTS:**

During 9,218 person-years of follow-up, the hypertension incidence rate was 30.6 (95% confidence interval [CI], 27.3 to 34.3) per 1,000 person-years. High job strain was associated with a higher risk for hypertension than low job strain (adjusted hazard ratio [aHR], 1.29; 95% CI, 1.00 to 1.67). High LTPA was associated with lower hypertension risk than low LTPA (aHR, 0.77; 95% CI, 0.60 to 0.98). Hypertension risk was higher among workers with high job strain and low LTPA than among those with low job strain and high LTPA (aHR, 1.70; 95% CI, 1.18 to 2.43).

**CONCLUSIONS:**

Job strain and LTPA showed positive and inverse associations, respectively, with incident hypertension. The combination of high job strain and low LTPA was associated with the highest risk for hypertension.

## INTRODUCTION

According to national statistics, the prevalence of hypertension, or high blood pressure, was 30-45% among United States adults [1,2] and 20-25% among United States workers during the past 2 decades [3,4]. Hypertension is one of the most decisive risk factors for cardiovascular diseases and is linked with higher cardiovascular-related and all-cause mortality [5,6]. Notably, the working-age mortality rate has increased in the United States since 2010, and hypertensive heart disease is one of the major drivers of rising mortality [7]. In addition, hypertension among United States workers is associated with productivity loss, including a substantial amount of short-term disability, absenteeism, and presenteeism [8]. Thus, the primary prevention of hypertension among United States workers has become a significant public health challenge.

For decades, job strain—a combination of high job demand and low job control—has been associated with elevated blood pressure and an increased risk for hypertension [9-12]. In contrast, leisure-time physical activity (LTPA) is a well-known modifiable factor in reducing blood pressure and hypertension risk, as evidenced by several interventional and observational studies [13-15] and the guideline published by the American College of Cardiology/American Heart Association [16].

A few epidemiological studies have investigated the joint associations of job strain and physical activity in the context of various health conditions. For example, large population-based observational studies among workers in Europe have demonstrated that LTPA could moderate the negative associations between job strain and health conditions, including sleep quality [17], depressive symptoms [18], and coronary heart disease [19,20]. Nonetheless, research on the joint associations between job strain and LTPA on the risk for hypertension is lacking. Therefore, the objective of this study was to examine the independent and joint associations of job strain and LTPA with incident hypertension in United States workers. To our knowledge, this is the first study to examine the joint associations of job strain and LTPA with incident hypertension using data from a national, population-based prospective cohort. We hypothesized that high job strain and low LTPA are independently associated with incident hypertension. We further hypothesized that high job strain and low LTPA are jointly associated with incident hypertension, such that workers with low job strain and high LTPA would have the lowest incidence of hypertension.

## MATERIALS AND METHODS

### Study sample

**Figure 1.**
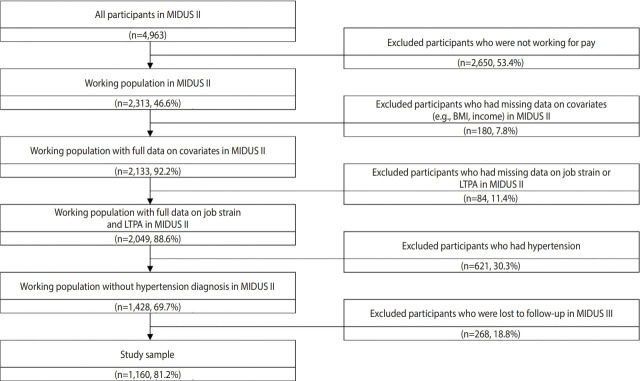
Sample selection flow chart. MIDUS, the Midlife in the United States; LTPA, leisure-time physical activity; BMI, body mass index.

The study participants were selected from the Midlife in the United States (MIDUS) II [21] and MIDUS III [22] surveys. The MIDUS study, initiated in 1994, is a national and longitudinal cohort study that focuses on the role of psychological, social, and biological factors for age-related variations in health and well-being among national samples of Americans. The MIDUS I survey did not collect comprehensive information on physical activity, so we did not use it in the current study. The MIDUS II survey collected data from 2004 to 2006, and MIDUS III collected data from 2013 to 2014. Data collection was primarily conducted through phone interviews using random-digit dialing and self-administered questionnaires. Among the total of 4,963 individuals who participated in the MIDUS II survey (baseline), 2,313 reported that they were working, of whom 2,049 workers without missing data on exposure variables or covariates were included. Among them, 1,428 workers had hypertension at baseline (defined by the question “Has a doctor ever told you that you have or had high blood pressure?”) and were excluded from this analysis, resulting in 1,160 workers who had follow-up data in the MIDUS III survey (Figure 1). The baseline characteristics of the 1,160 workers and 268 workers who were lost to follow-up were compared to assess potential selection bias (Supplementary Material 1) .

### Exposures

Job strain was derived from Karasek’s job demand-control model and defined as the combination of high job demands and low job control [23]. At baseline, job strain was operationalized based on questions regarding job demands and job control (i.e., the sum of decision authority and skill discretion). Job demands and job control were dichotomized into high and low groups by median scores (15 and 34, respectively). For job strain (binary), high job demands and low job control were combined to form the high strain group, and other participants were dichotomized into the low strain group. This approach to defining binary job strain has been justified and applied by large epidemiological investigations with international multi-cohort data [24,25], as well as a previous publication using the MIDUS data [23]. In a sensitivity analysis, job strain was also classified into 4 categories following the traditional quadrant approach: low strain (low demand+high control), active (high demand+high control), passive (low demand+low control), and high strain (high demand+low control) [26].

Moderate-to-vigorous LTPA at baseline was assessed with questions (4 items) related to LTPA frequency. The responses were measured via a 6-point scale (1= several times a week, 6= never). Vigorous LTPA was defined as an activity that caused a rapid heartbeat and heavy sweat. Moderate LTPA was defined as an activity that was not physically exhausting but caused the heart rate to increase slightly and induced a light sweat. Moderate LTPA and vigorous LTPA in summer and winter were averaged, respectively. Then, workers who reported performing moderate LTPA or vigorous LTPA at least once per week were classified as engaging in high moderate-to-vigorous LTPA, and the others were classified as engaging in low moderate-to-vigorous LTPA. Moderate LTPA and vigorous LTPA were combined because the American Heart Association guideline recommends performing at least 150 min/wk of moderate aerobic activity or 75 min/wk of vigorous aerobic activity, or a combination of both, to enhance health for adults [27]. This approach to LTPA measurement has been applied in a previous MIDUS publication [28].

### Outcome

Hypertension was self-reported by the question “Has a doctor ever told you that you have or had high blood pressure?” at follow-up. The timing of the hypertension diagnosis in years since the baseline survey was self-reported via the question: “How many years ago were you told you have or had high blood pressure?” at follow-up. We defined incident hypertension as a new diagnosis of hypertension during the follow-up period among those without prevalent hypertension at baseline. This approach has been widely used in other national studies in the United States, such as the Health and Retirement Study [29]. The current study observed incident hypertension from 2004-2006 (MIDUS II) to 2013-2014 (MIDUS III). The hypertension diagnosis criteria (systolic blood pressure [SBP] ≥ 140 mmHg, diastolic blood pressure ≥ 90 mmHg) published in 2003 were likely applied during the study period [30].

### Covariates

Data on socio-demographic factors and health-related behaviors were collected at baseline. Covariates were pre-selected: age (continuous), sex (male, female), marital status (married, never married, others), race (White, non-White), educational attainment (high school or less, some college, bachelor’s degree or more), annual household income (< US$60,000, 60,000-99,999, ≥ 100,000), body mass index (BMI) (normal, overweight, obese), smoking (yes, no), heavy alcohol drinking (yes [> 2 drinks per day for males and > 1 drink per day for females], no) [28,31]. These potential confounders were selected as they have been found to be associated with job strain and LTPA, and they are known risk factors for hypertension [32-34].

### Statistical analysis

At baseline, frequency (percentage) for categorical covariates and mean (standard deviations, SD) for continuous covariates were calculated. Statistical differences in covariates between the job strain and LTPA groups were compared using the two-sample t-test or the chi-square test.

The incidence of hypertension and 95% confidence intervals (CIs) during the follow-up period were estimated. The incidence of hypertension by job strain and LTPA groups was displayed using Kaplan–Meier survival curves. Events were defined at the time of reported hypertension onset. Participants were censored upon hypertension diagnosis or at the end of follow-up.

The associations of job strain and LTPA with incident hypertension were illustrated in directed acyclic graphs (Supplementary Material 2). In this study, baseline job strain and LTPA were not correlated (chi-square test, p= 0.75); furthermore, job strain and LTPA were measured at the same time, resulting in a lack of a temporal relationship. Thus, we did not consider the mediating effect of LTPA on the association between job strain and incident hypertension. Cox proportional hazards models were used to estimate hazard ratios (HRs) and 95% CIs. The proportional hazards assumptions were verified prior to model selection. Following the unadjusted model, the adjusted model was calculated by adjusting for socio-demographic factors (age, sex, marital status, and race), socioeconomic position (educational attainment and annual household income), and behavioral factors (BMI, smoking status, and alcohol consumption). Independent associations of job strain and LTPA with incident hypertension were estimated with mutual adjustment for job strain and LTPA. Joint associations of job strain and LTPA with incident hypertension were estimated by creating a composite variable with different combinations of job strain and LTPA (i.e., low job strain, high LTPA [HR_00_ reference]; high job strain, high LTPA [HR10]; low job strain, low LTPA [HR01]; high job strain, low LTPA [HR_11_]). The synergy index, (HR_11_–1)/([HR_01_-1]+[HR_10_-1]), was calculated. A synergy index greater than 1 indicates a synergistic interaction, equal to 1 indicates an additive interaction, and less than 1 indicates an antagonistic interaction [35]. The multiplicative interaction term of job strain and LTPA with incident hypertension was also examined.

All analyses were conducted using SAS version 9.4 (SAS Institute Inc., Cary, NC, USA). A 2-sided p-value less than 0.05 was considered statistically significant.

### Ethics statement

This study was reviewed and approved for exemption by the University of California, Los Angeles Institutional Review Board (IRB#22-000604). Written informed consent was collected from all participants.

## RESULTS

### Baseline characteristics

In total, 838 (72.2%) and 322 (27.8%) participants were classified as having low or high job strain, respectively. The mean± SD age was 50.08± 9.30 years in the low job strain group, which was older than that of 48.61± 8.15 years in the high job strain group. Participants in the low job strain group were more likely to be males (low: 50.0 vs. high: 42.5%), normal weight (42.0 vs. 37.6%), and heavy alcohol drinkers (2.7 vs. 0.6%) than those in the high job strain group.

Furthermore, 528 (45.5%) and 632 (54.5%) participants were classified as engaging in low or high LTPA, respectively. The mean age was 50.25± 9.08 years in the low LTPA group, which was older than that of 49.19± 8.94 years in the high LTPA group. The participants in the low LTPA group were less likely to be normal weight (36.7 vs. 44.1%), White (91.9 vs. 95.4%), have a bachelor’s degree or more (42.6 vs. 58.2%), and have an annual household income of at least US$100,000 (28.2 vs. 39.1%), while they were more likely to be smokers (18.7 vs. 8.7%) (Table 1).

### Hypertension incidence

**Figure 2.**
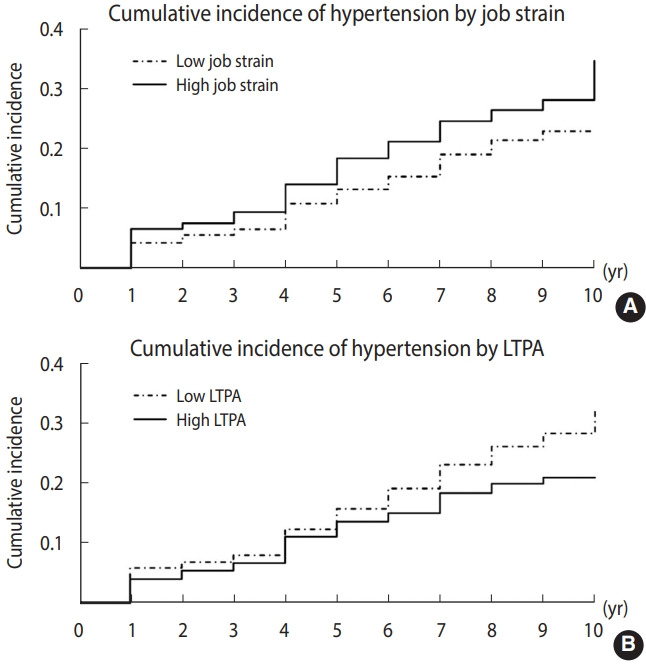
Kaplan-Meier cumulative incidence curves for hypertension by baseline job strain (A) and leisure-time physical activity (LTPA) (B).

During a mean of 8 years (9,218 person-years) of follow-up, 282 new hypertension cases were reported. The overall incidence rate of hypertension was 30.59 (95% CI, 27.27 to 34.31) per 1,000 person-years at the end of follow-up. The Kaplan-Meier curves clearly indicated that the high job strain group had a higher incidence of hypertension than the low job strain group, and the low LTPA group had a higher incidence of hypertension than the high LTPA group (Figure 2, Supplementary Material 3).

### Independent associations of baseline job strain and leisure-time physical activity with incident hypertension

The incidence rates of hypertension in the participants with low job strain, high job strain, low LTPA, and high LTPA were 28.16 (95% CI, 24.48 to 32.38), 36.69 (95% CI, 29.99 to 44.89), 36.28 (95% CI, 31.01 to 42.46), and 25.96 (95% CI, 21.94 to 30.72) per 1,000 person-years, respectively. The risk of hypertension was higher in the high job strain group than in the low job strain group (unadjusted HR, 1.29; 95% CI, 1.01 to 1.66; adjusted HR, 1.29; 95% CI, 1.00 to 1.67). The risk of hypertension was lower in the high LTPA group than in the low LTPA group (unadjusted HR, 0.72; 95% CI, 0.57 to 0.91; adjusted HR, 0.77; 95% CI, 0.60 to 0.98). The results are nearly unchanged after adjusting for potential confounders (Table 2). In the sensitivity analysis with job strain classified into 4 categories, there was no obviously different pattern in the “active” and “passive” groups compared to the “low strain” group, lending support to our binary approach to defining job strain in the main analyses (Supplementary Material 4).

### Joint associations of baseline job strain and leisure-time physical activity with incident hypertension

The incidence rates of hypertension were 23.91 (95% CI, 19.47 to 29.35) per 1,000 person-years in the low job strain and high LTPA group, 31.59 (95% CI, 23.54 to 42.40) per 1,000 person-years in the high job strain and high LTPA group, 33.83 (95% CI, 27.96 to 40.94) per 1,000 person-years in the low job strain and low LTPA group, and 42.90 (95% CI, 32.52 to 56.57) per 1,000 person-years in the high job strain and low LTPA group. In the unadjusted model, compared with the low job strain and high LTPA group (reference), the risk of hypertension was higher in the low job strain and low LTPA group (HR, 1.41; 95% CI, 1.06 to 1.87) and the high job strain and low LTPA group (HR, 1.79; 95% CI, 1.26 to 2.54). In the adjusted model, compared with the low job strain and high LTPA group, the risk of hypertension was significantly elevated in the high job strain and low LTPA group (HR, 1.70; 95% CI, 1.18 to 2.43). The synergy index was not significantly different from 1 (unadjusted: HR, 1.08; 95% CI, 0.41 to 2.88; adjusted: HR, 1.20; 95% CI, 0.39 to 3.73), indicating an additive interaction between job strain and LTPA. The multiplicative interaction term of job strain and LTPA with incident hypertension was also not significant (unadjusted p-value, 0.89; adjusted p-value, 0.94). The results remains robust after adjusting for potential confounders (Table 3).

## DISCUSSION

In this prospective cohort study with a mean of 8 years of follow-up, we found that high job strain and low LTPA were independently associated with an elevated risk for hypertension. When analyzing the joint associations between job strain and LTPA, the high job strain and low LTPA group had a nearly 2-fold higher risk for hypertension than the reference group (low job strain and high LTPA). An additive interaction between job strain and LTPA was observed. In general, high LTPA could reduce the risk of incident hypertension among workers, regardless of their job strain level, by 24%. Specifically, among workers with low or high job strain, high LTPA was associated with a lower hypertension risk by 23% or 25%, respectively. Therefore, our hypotheses were supported by our findings.

The reported independent associations between job strain, LTPA, and incident hypertension are wholly consistent with the previous literature. For example, a meta-analysis of observational studies found that the pooled risk estimates of job strain for incident hypertension based on cohort research design was 1.24 (95% CI, 1.09 to 1.41) [10]. In a meta-analysis of prospective cohort studies, moderate and vigorous LTPA were associated with decreased hypertension risk compared to low LTPA (risk ratio, moderate vs. low: 0.89; 95% CI, 0.85 to 0.94; high vs. low: 0.81; 95% CI, 0.76 to 0.85) [15]. Although joint associations between job strain, LTPA, and incident hypertension have not been systematically investigated previously, our findings are in line with empirical evidence substantiating joint associations of job strain and physical activity on various health conditions, including sleep quality, depressive symptoms, and coronary heart diseases [17-20]. For instance, a large cross-sectional study in Sweden demonstrated that 42% of workers who had high job strain and low physical activity self-reported poor sleep quality, compared to 23% of workers who had low job strain and high physical activity [17]. In a large cohort study from 2001 to 2007 in Finland, participation in LTPA reduced the detrimental effects of job strain on incident depressive symptoms among female workers [18]. A pooled analysis of several European cohort studies reported that the 10-year incidence of coronary artery disease was the highest among workers with high job strain and unhealthy lifestyles (i.e., physical inactivity, smoking, heavy drinking, and obesity) [19]. Another large cohort study among male workers in Italy reported that after 14 years of follow-up, coronary heart disease risk was high among participants with high job strain, while LTPA could attenuate the risk among non-manual heavy workers [20].

The mechanistic pathways underlying the independent and joint associations of job strain and LTPA with incident hypertension may be complex, spanning interactions of cardiometabolic and neuroendocrine biology. Job strain is a well-evidenced psychosocial stressor that contributes to persistent elevations of blood pressure [36]. LTPA may reduce blood pressure via multiple circulatory and biomolecular pathways, including reduction of vascular resistance [37], anti-inflammatory metabolic cascades [38,39], weight loss [40], and improved insulin sensitivity [41]. The additive effect of job strain and LTPA on incident hypertension suggests that the mechanistic pathways for job strain and LTPA with incident hypertension may be neuroanatomically unique or otherwise potentiated.

This study represents an expansion of the potential means by which modifiable risk factors (job strain and LTPA) for hypertension can be leveraged to inform government and employer policy changes or workplace health interventions. For example, in a quasi-experimental study in Canada, the prevalence of hypertension (prevalence ratio: 0.85; 95% CI, 0.74 to 0.98) and SBP (-2.0 mmHg; 95% CI, -3.0 to -1.0) was lower in the intervention group (i.e., organizational changes to reduce levels of work stress, including job strain, for 17 to 24 months) than in the control group [42]. A large randomized controlled trial in the workplace in Canada found that office workers who adhered to a physical exercise training program for 1 year (1 hour of supervised high-intensity training every week within working hours and recommendations for 30 minutes of moderate LTPA 6 days a week) had lower SBP (-3.16 mmHg; 95% CI, -6.22 to -0.10) than controls [43]. Thus, the combination of stress management and LTPA promotion programs at work is expected to lead to an additive reduction in blood pressure and incident hypertension.

This study has several unique strengths. First, the MIDUS was a large, national study that included a diverse range of occupations, increasing the generalizability of the results. Second, this study utilized reliable and valid measures for exposure variables. Job strain was defined using established scales [23], and LTPA was determined based on a national guideline [27]. Lastly, potential confounders, including socio-demographic factors, lifestyle behaviors, and BMI, were collected and controlled.

This study has a few potential limitations. First, this is an observational study. Although we carefully controlled for potential confounders, residual confounding may exist. Second, hypertension was self-reported, which could have led to an underestimation of incidence [44]. Third, job strain and LTPA were measured at baseline, although they might have changed over time. Repeated measures of job strain and LTPA may improve exposure ascertainments [34,35]. Finally, approximately 20% of participants were lost to follow-up in MIDUS III, leading to potential selection bias. They were less likely to be well-educated and married, and more likely to be smokers, than the study sample. Regardless, they were similar to the study sample in the majority of characteristics at the time of MIDUS II, including age, sex, race, annual household income, BMI, and alcohol consumption (Supplementary Material 1).

In conclusion, this study demonstrated that high job strain and low LTPA were independent risk factors for hypertension in a large sample of middle-aged United States workers. The combined exposure to high job strain and low LTPA resulted in the highest risk for hypertension. In the face of mounting challenges to public health due to the devastating and persistent disease and mortality burden of hypertension and cardiovascular diseases, it is critical that government and employer policy interventions target job strain and LTPA as potentially modifiable risk factors for the goal of hypertension reduction.

## Figures and Tables

**Table 1.** Baseline characteristics at MIDUS II

Characteristics		Job strain			p-value^1^	LTPA		p-value^1^
		Overall (n=1,160)	Low (n=838)	High (n=322)		Low (n=528)	High (n=632)	
Age, mean±SD (yr)		49.67±9.02	50.08±9.30	48.61±8.15	0.01	50.25±9.08	49.19±8.94	0.05
Male		556 (47.9)	419 (50.0)	137 (42.5)	0.02	259 (49.0)	297 (47.0)	0.48
BMI (kg/m^2^)					0.01			<0.001
	Normal (<25.0)	473 (40.8)	352 (42.0)	121 (37.6)		194 (36.7)	279 (44.1)	
	Overweight (25.0-29.9)	431 (37.2)	320 (38.2)	111 (34.5)		188 (35.6)	243 (38.4)	
	Obese (≥30.0)	256 (22.1)	166 (19.8)	90 (27.9)		146 (27.6)	110 (17.4)	
Married		871 (75.1)	641 (76.5)	230 (71.4)	0.07	400 (75.8)	471 (74.5)	0.63
White		1,088 (93.8)	788 (94.0)	300 (93.2)	0.58	485 (91.9)	603 (95.4)	0.01
Education					0.13			<0.001
	High school or less	256 (22.1)	184 (22.0)	72 (22.4)		155 (29.4)	101 (16.0)	
	Some college	311 (26.8)	212 (25.3)	99 (30.7)		148 (28.0)	163 (25.8)	
	Bachelor’s degree or more	593 (51.1)	442 (52.7)	151 (46.9)		225 (42.6)	368 (58.2)	
Annual household income (US$)					0.12			<0.001
	<60,000	392 (33.8)	276 (32.9)	116 (36.0)		199 (37.7)	193 (30.5)	
	60,000-99,999	372 (32.1)	261 (31.1)	111 (34.5)		180 (34.1)	192 (30.4)	
	≥100,000	396 (34.1)	301 (35.9)	95 (29.5)		149 (28.2	247 (39.1)	
Current smoking		154 (13.3)	111 (13.2)	43 (13.3)	0.96	99 (18.7)	55 (8.7)	<0.001
Current heavy alcohol drinking		25 (2.2)	23 (2.7)	2 (0.6)	0.03	15 (2.8)	10 (1.6)	0.14

Values are presented as number (%). MIDUS, the Midlife in the United States; LTPA, leisure-time physical activity; SD, standard deviation; BMI, body mass index.

1 Two-sample t-tests were used to compare continuous variables; Chi-squared tests were used to compare categorical variables.

**Table 2.** Independent associations of baseline job strain and LPTA with incident hypertension (n=1,160)

Variables	Per 1,000 person-yr	Unadjusted model	Adjusted model^1^
	IR (95% CI)	HR (95% CI)^2^	HR (95% CI)^2^
Job strain			
Low	28.16 (24.48, 32.38)	1.00 (reference)	1.00 (reference)
High	36.69 (29.99, 44.89)	1.29 (1.01, 1.66)^*^	1.29 (1.00, 1.67)^*^
LTPA			
Low	36.28 (31.01, 42.46)	1.00 (reference)	1.00 (reference)
High	25.96 (21.94, 30.72)	0.72 (0.57, 0.91)^*^	0.77 (0.60, 0.98)^*^

LTPA, leisure-time physical activity; IR, incidence rate; CI, confidence interval; HR, hazard ratio.

1 Adjusted model controlled for age, sex, marital status, race, education, annual household income, body mass index, smoking, and heavy alcohol drinking.

2 Cox proportional hazard models were used to estimate the HR and 95% CI.

* p<0.05.

**Table 3.** Joint associations of baseline job strain and LTPA with incident hypertension (n=1,160)

Variables	Per 1,000 person-yr	Unadjusted model	Adjusted model^1^
	IR (95% CI)	HR (95% CI)^2^	HR (95% CI)^2^
Job strain			
Low, LTPA high (HR_00_)	23.91 (19.47, 29.35)	1.00 (reference)	1.00 (reference)
High, LTPA high (HR_01_)	31.59 (23.54, 42.40)	1.32 (0.91, 1.89)	1.28 (0.89, 1.85)
Low, LTPA low (HR_10_)	33.83 (27.96, 40.94)	1.41 (1.06, 1.87)^*^	1.30 (0.97, 1.74)
High, LTPA low (HR_11_)	42.90 (32.52, 56.57)	1.79 (1.26, 2.54)^*^	1.70 (1.18, 2.43)^*^
Synergy index (HR_11_-1)/([HR_01_-1]+[HR_10_-1])	-	1.08 (0.41, 2.88)	1.20 (0.39, 3.73)

LTPA, leisure-time physical activity; IR, incidence rate; CI, confidence interval; HR, hazard ratio.

1 Adjusted model controlled for age, sex, marital status, race, education, annual household income, body mass index, smoking, and heavy alcohol drinking.

2 Cox proportional hazard models were used to estimate the HR and 95% CI.

* p<0.05.

## SUPPLEMENTARY MATERIALS

Supplementary Material 1. Baseline characteristics for the study sample and those who were lost to follow-up in the study at MIDUS II

Supplementary Material 2. Directed acyclic graphs (DAGs) for the associations of baseline job strain and leisure-time physical activity with incident hypertension

Supplementary Material 3. Kaplan-Meier cumulative incidence curves for hypertension by baseline job strain and leisure-time physical activity

Supplementary Material 4. Independent associations of baseline job strain (4 categories) and leisure-time physical activity with incident hypertension
