# Supplementary material for: The associations of job strain and leisure-time physical activity with the risk of hypertension: the population-based Midlife in the United States cohort study
Source: Epidemiol Health. 2022 Sep 7;44:e2022073. doi: 10.4178/epih.e2022073 (PMC9849846; doi:10.4178/epih.e2022073)
Supplement: Supplementary Material 1. — Baseline characteristics for the study sample and those who were lost to follow-up in the study at MIDUS II [file epih-44-e2022073-suppl1.docx]

**Supplementary Material 1.** Baseline characteristics for the study sample and those who were lost to follow-up in the study at MIDUS II

|  | **Study sample** | | **Lost to follow-up** | | **P-value** |
| --- | --- | --- | --- | --- | --- |
|  | **N = 1,160** | | **N = 268** | |  |
| Age, mean (SD) | 49.67 | (9.02) | 48.59 | (9.70) | 0.08 |
| Male, N (%) | 556 | (47.93) | 128 | (47.76) | 0.96 |
| BMI category, N (%) |  |  |  |  | 0.07 |
| Normal (<25) | 473 | (40.78) | 97 | (36.19) |  |
| Overweight (25-29.9) | 431 | (37.16) | 120 | (44.78) |  |
| Obese (≥30) | 256 | (22.07) | 51 | (19.03) |  |
| Married, N (%) | 871 | (75.09) | 184 | (68.66) | 0.03* |
| White, N (%) | 1,088 | (93.79) | 247 | (92.16) | 0.33 |
| Education, N (%) |  |  |  |  | <0.0001* |
| High school or less | 256 | (22.07) | 84 | (31.34) |  |
| Some college | 311 | (26.81) | 90 | (33.58) |  |
| Bachelor’s degree or more | 593 | (51.12) | 94 | (35.07) |  |
| Annual household Income, N (%) |  |  |  |  | 0.36 |
| <$60,000 | 392 | (33.79) | 103 | (38.43) |  |
| $60,000-$99,999 | 372 | (32.07) | 80 | (29.85) |  |
| ≥$100,000 | 396 | (34.14) | 85 | (31.72) |  |
| Current smoking, N (%) | 154 | (13.28) | 58 | (21.64) | 0.0005* |
| Current heavy alcohol drinking, N (%) | 25 | (2.16) | 3 | (1.12) | 0.27 |
| *Abbreviation: BMI, body mass index; LTPA, leisure-time physical activity; MIDUS, the Midlife in the United States; SD, standard deviation.* | | | | | |

| Notes:  1. Two-sample t-tests were used to compare continuous variables; Chi-squared tests were used to compare categorical variables.  *P<0.05. |
| --- |
