# Supplementary material for: The associations of job strain and leisure-time physical activity with the risk of hypertension: the population-based Midlife in the United States cohort study
Source: Epidemiol Health. 2022 Sep 7;44:e2022073. doi: 10.4178/epih.e2022073 (PMC9849846; doi:10.4178/epih.e2022073)
Supplement: Supplementary Material 2. — Directed acyclic graphs (DAGs) for the associations of baseline job strain and leisure-time physical activity with incident hypertension [file epih-44-e2022073-suppl2.docx]

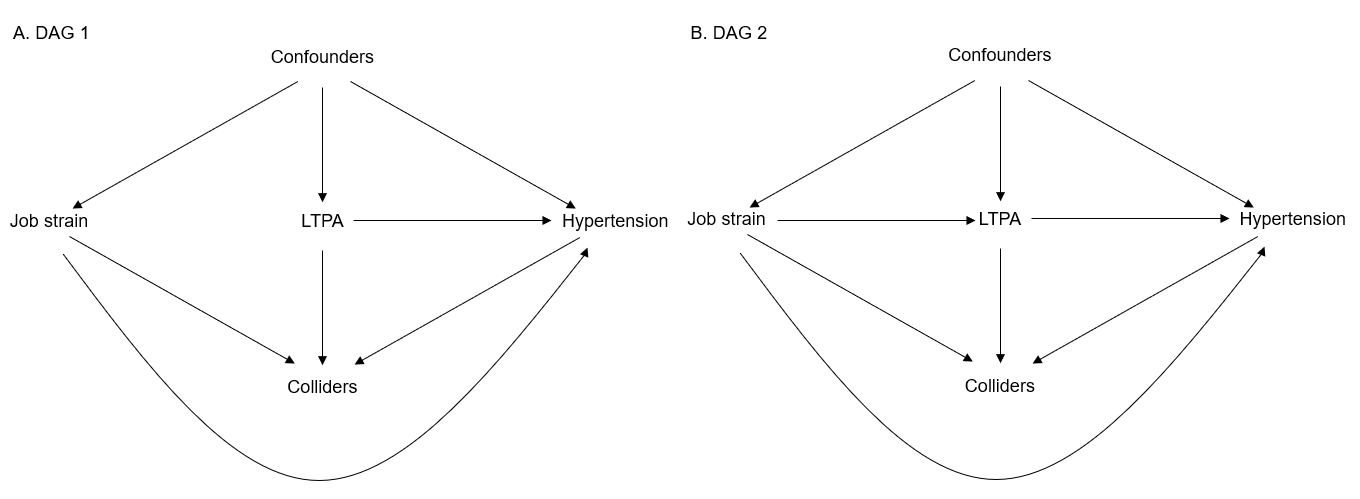
 *Abbreviations: DAG, directed acyclic graph; LTPA, leisure-time physical activity.*

**Supplementary Material 2.** Directed acyclic graphs (DAGs) for the associations of baseline job strain and leisure-time physical activity with incident hypertension
