# Supplementary material for: The associations of job strain and leisure-time physical activity with the risk of hypertension: the population-based Midlife in the United States cohort study
Source: Epidemiol Health. 2022 Sep 7;44:e2022073. doi: 10.4178/epih.e2022073 (PMC9849846; doi:10.4178/epih.e2022073)
Supplement: Supplementary Material 3. — Kaplan-Meier cumulative incidence curves for hypertension by baseline job strain and leisure-time physical activity [file epih-44-e2022073-suppl3.docx]

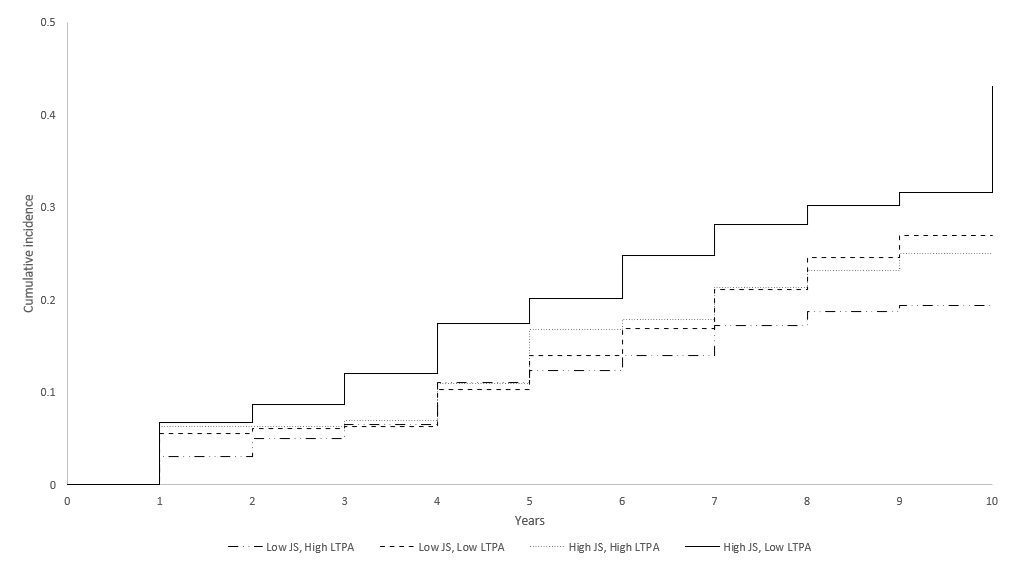


*Abbreviations: LTPA, leisure-time physical activity; JS, job strain.*

**Supplemental Material 3.** Kaplan-Meier cumulative incidence curves for hypertension by baseline job strain and leisure-time physical activity
