# Supplementary material for: The associations of job strain and leisure-time physical activity with the risk of hypertension: the population-based Midlife in the United States cohort study
Source: Epidemiol Health. 2022 Sep 7;44:e2022073. doi: 10.4178/epih.e2022073 (PMC9849846; doi:10.4178/epih.e2022073)
Supplement: Supplementary Material 4. — Independent associations of baseline job strain (4 categories) and leisure-time physical activity with incident hypertension [file epih-44-e2022073-suppl4.docx]

|  |
| --- |

**Supplementary Material 4.** Independent associations of baseline job strain (4 categories) and leisure-time physical activity with incident hypertension

| **N = 1,160** | **Per 1,000 Person-years** | | | **Model I** | | | **Model II** | | | **Model III** | | | **Model IV** | | |
| --- | --- | --- | --- | --- | --- | --- | --- | --- | --- | --- | --- | --- | --- | --- | --- |
|  | **IR** | **95% CI** | | **HR** | **95% CI** | | **HR** | **95% CI** | | **HR** | **95% CI** | | **HR** | **95% CI** | |
| Low job strain | 28.46 | (21.59 | 37.50) | 1 |  |  | 1 |  |  | 1 |  |  | 1 |  |  |
| Passive | 27.83 | (22.06 | 35.12) | 0.96 | (0.67 | 1.39) | 0.99 | (0.68 | 1.43) | 0.96 | (0.66 | 1.40) | 0.98 | (0.67 | 1.42) |
| Active | 28.77 | (22.95 | 36.07) | 1.03 | (0.71 | 1.47) | 1.13 | (0.78 | 1.64) | 1.15 | (0.79 | 1.66) | 1.13 | (0.78 | 1.64) |
| High job strain | 36.69 | (29.99 | 44.89) | 1.28 | (0.91 | 1.82) | 1.39 | (0.98 | 1.99) | 1.36 | (0.95 | 1.94) | 1.34 | (0.94 | 1.92) |
| Low LTPA | 36.28 | (31.01 | 42.46) | 1 |  |  | 1 |  |  | 1 |  |  | 1 |  |  |
| High LTPA | 25.96 | (21.94 | 30.72) | 0.72* | (0.57 | 0.90) | 0.73* | (0.58 | 0.93) | 0.75* | (0.59 | 0.95) | 0.76* | (0.60 | 0.97) |
| *Abbreviations: BMI, body mass index; CI, confidence interval; HR, hazard ratio; IR, incidence rate; LTPA, leisure-time physical activity.* | | | | | | | | | | | | | | | |
| Notes:  1. Cox proportional hazard models were used to estimate the HR and 95% CI.  2. Model I: unadjusted; Model II: adjusted for sociodemographic factors (age, sex, marital status, and race); Model III: Model II + additionally adjusted for socioeconomic positions (educational attainment and annual household income); Model IV: Model III + additionally adjusted for behavioral factors (BMI, smoking, and heavy alcohol drinking).  *P-value<0.05. | | | | | | | | | | | | | | | |
